# Establishing and validation of the VBV score for assessing Lung ground-glass nodules based on high-resolution computed tomography

**DOI:** 10.1186/s13019-024-02487-3

**Published:** 2024-01-23

**Authors:** Yuwei Zhou, Xiaoqing Cao, Haiyong Gu, Shenhu Gao, Yuxuan Wu, Haoyang Li, Bing Xiong, Haiyang Dong, Yan Lv, Rong Yang, Yihe Wu

**Affiliations:** 1 Department of Thoracic Surgery, the First Affiliated Hospital, Zhejiang University School of Medicine, 79 Qingchun Road, Hangzhou, 310003 China; 2 Department of Thoracic Surgery, Ningbo Medical Center Lihuili Hospital, Ningbo, Zhejiang China; 3 Department of Thoracic Surgery, Beijing Chest Hospital, Capital Medical University, Beijing Tuberculosis and Thoracic Tumor Research Institute, Beijing, China; 4 Department of Thoracic Surgery, Shanghai Chest Hospital, School of Medicine, Shanghai Jiao Tong University, Shanghai, China; 5 Department of Radiology, the First Affiliated Hospital, Zhejiang University School of Medicine, Hangzhou, China; 6 Department of Radiology, Shanghai Chest Hospital, School of Medicine, Shanghai Jiao Tong University, Shanghai, China; 7 Department of Medical Imaging, Beijing Chest Hospital, Capital Medical University, Beijing Tuberculosis and Thoracic Tumor Research Institute, Beijing, China

**Keywords:** Lung cancer, Computed tomography, Ground-glass nodules, Risk score

## Abstract

**Background:**

The widespread utilization of chest High-resolution Computed Tomography (HRCT) has prompted detection of pulmonary ground-glass nodules (GGNs) in otherwise asymptomatic individuals. We aimed to establish a simple clinical risk score model for assessing GGNs based on HRCT.

**Methods:**

We retrospectively analyzed 574 GGNs in 574 patients undergoing HOOK-WIRE puncture and pulmonary nodule surgery from January 2014 to November 2018. Clinical characteristics and imaging features of the GGNs were assessed. We analyzed the differences between malignant and benign nodules using binary logistic regression analysis and constructed a simple risk score model, the VBV Score, for predicting the malignancy status of GGNs. Then, we validated this model via other 1200 GGNs in 1041 patients collected from three independent clinical centers in 2022.

**Results:**

For the exploratory phase of this study, out of the 574 GGNs, 481 were malignant and 93 were benign. Vacuole sign, air bronchogram, and intra-nodular vessel sign were important indicators of malignancy in GGNs. Then, we derived a VBV Score = vacuole sign + air bronchogram + intra-nodular vessel sign, to predict the malignancy of GGNs, with a sensitivity, specificity, and accuracy of 95.6%, 80.6%, and 93.2%, respectively. We also validated it on other 1200 GGNs, with a sensitivity, specificity, and accuracy of 96.0%, 82.6%, and 95.0%, respectively.

**Conclusions:**

Vacuole sign, air bronchogram, and intra-nodular vessel sign were important indicators of malignancy in GGNs. VBV Score showed good sensitivity, specificity, and accuracy for differentiating benign and malignant pulmonary GGNs.

**Supplementary Information:**

The online version contains supplementary material available at 10.1186/s13019-024-02487-3.

## Introduction

Lung cancer is one of the most frequently diagnosed cancers and the leading cause of cancer-related deaths worldwide [1]. Early diagnosis and treatment are effective methods for improving the survival rate of lung cancer patients. High-resolution computed tomography (HRCT) can find small nodules in the lung and is the most effective diagnostic tool for early lung cancer [2]. According to the different density of pulmonary nodules in CT images, nodules can be divided into pure ground-glass nodules (GGNs), partially solid nodules, and solid nodules [3]. In HRCT, GGNs are hazy and amorphous areas with increased attenuation of the lung that do not obscure underlying bronchial structures or vascular margins, which frequently turned out to be early-stage lung cancer [4]. In addition, benign lesions, such as inflammation, pulmonary edema, and pulmonary thromboembolism, can also present as GGNs [5]. Analyzing CT image features of GGNs is the key to distinguish benign and malignant GGNs, which affects the choice of clinical treatment methods [6]. At present, there is no uniform standard for judging GGNs, and clinicians often make judgments based on experience, which may lead to misdiagnosis and missed diagnosis. However, in many large thoracic surgery clinical centers, including our center, the coincidence rate of preoperative diagnosis and postoperative pathology of thoracic surgery can reach more than 90%, indicating that the accuracy rate of experienced thoracic surgeons in judging GGNs is dearly high [7]. To sum up the experience of thoracic surgeons and radiologists in judging whether GGN is benign or malignant, and to establish a simple and practical model is of great clinical significance for the diagnosis and treatment of GGNs.

The purpose of the current study was to establish a simple clinical risk score model for assessing GGNs on the basis of morphological features of pathologically confirmed GGNs on HRCT and determine the validity of this scoring system. This scoring system will be used to help clinicians judge whether GGN is benign or malignant, and then take appropriate treatment strategies.

## Patients and methods

### Patient information

In the current study, our institution retrospectively identified 600 patients undergoing pulmonary nodule surgery and went through their preoperative CT images and pathology records at the Department of Thoracic Surgery, the First Affiliated Hospital, School of Medicine, Zhejiang University between January 2014 to November 2018. All the patients underwent preoperative CT-assisted HOOK-WIRE puncture to locate the nodules before the pulmonary nodule surgery. The reason for choosing HOOK-WIRE patients is to facilitate nodule location at surgery,. 26 patients were excluded from the study for the following reasons: (1) incomplete clinical and pathological information or CT imaging data (*n* = 14); (2) the maximum diameter of the nodule exceeds 3 cm (*n* = 5); (3) the nodules were completely solid nodules and mean attenuation value was large than 0 (*n* = 7). Finally, we confirmed 574 GGNs resected from 574 patients for the present study. Clinical and pathologic information was obtained from medical records, including age, gender, follow-up time, histories of malignant tumors, and smoking history. According to the same standard, we additionally collected 1200 GGNs in 1041 patients from three clinical centers starting from January 1, 2022 for validation. Among them, 600 GGNs in 499 patients came from Department of Thoracic Surgery, the First Affiliated Hospital, School of Medicine, Zhejiang University between January 1 and April 26; 300 GGNs in 272 patients came from Department of Thoracic Surgery, Shanghai Chest Hospital Shanghai Jiaotong University between January 1 and February 15, 300 GGNs in 270 patients from Department of Thoracic Surgery, Beijing Chest Hospital, Capital Medical University between January 1 and March 25. The Ethics Committee of each participating center approved this study. Informed consent was waived because this was a retrospective study.

### CT image acquisition

All the patients were subjected to HRCT analysis during breath holding at mid-inspiration using a Lightspeed CT system (General Electric Healthcare, Milwaukee WI, USA) or Brilliance CT 64 (Philips Medical Systems, Best, the Netherlands). The scanning parameters were as follows: 120 mA, tube voltage of 120 kV, and a field of view of 36.0 cm. The reconstruction thicknesses and intervals were 1.0 or 1.25 mm.

### Evaluation of CT characteristics

All the post-processed images were reviewed by a thoracic surgeon and a radiologist with 10 years of experience in chest CT. They analyzed and discussed the Digital Imaging and Communications in Medicine (DICOM) images from HRCT. The observers were blinded to the subjects’ identities and clinical data. Decisions regarding CT findings were made via consensus. The characteristics of GGNs were assessed according to the nodule location, shape, presence of lobulation, spinous process, vacuole sign, air bronchogram, intra-nodular vessel sign, calcification, fat, necrosis, and pleural indentation. The nodule location was mainly represented by the lobe, such as the right upper lobe (RUL), etc. Nodule size was defined as the maximal diameter of the nodule. Nodule shape was classified as round/oval or irregular. Lobulation was considered to present as nodules showing different or uneven growth rates. Spinous process refers to the angular or columnar protrusions that can be seen on the edge of the nodule. Vacuole sign was defined as a gas-filled space within the GGNs [8, 9]. Air bronchogram were defined as air-containing bronchi or bronchioles within the GGNs [10]. Intra-nodular vessel sign refers to that one or more vessels can be seen passing through the GGNs [11]. Pleural indentation was considered to present via an attachment to the visceral pleura surface with the nodule [12].

### Pathological diagnoses

All pathological specimens were obtained via video-assisted thoracic surgery (VATS) or open thoracotomy. Pathological diagnoses of the 574 GGNs were based on the criteria of the 2011 IASLC/ATS/ERS and 2015 WHO classifications [13, 14]. For the validation set, the pathological diagnoses were based on the criteria of the 2021 WHO classification [15].

### Statistical analysis

Quantitative data are presented as the mean ± standard deviation, whereas categorical data are presented as numbers and percentages. Analyses of clinical characteristics and CT morphological features between benign and malignant GGNs were performed using the Chi-square test. Patients’ age and the maximum diameter of GGNs were analyzed with the Mann–Whitney U-test. Additionally, the significant characteristics were analyzed using binary logistic regression analysis via the forward likelihood ratio (LR) method to establish a new score model, VBV Score. Diagnostic concordance was determined by calculating the Kappa Coefficient. Then, VBV Score was validated via the three independent dataset by determining each GGNs’ score and evaluating the diagnostic accuracy and diagnostic concordance. A two-sided *p* value < 0.05 was considered to indicate statistical significance. All statistical analyses were performed using SPSS version v.11.0 software (SPSS Inc., Chicago, IL).

## Results

### Comparison of clinical characteristics and morphological features between benign and malignant GGNs

**Fig. 1:**
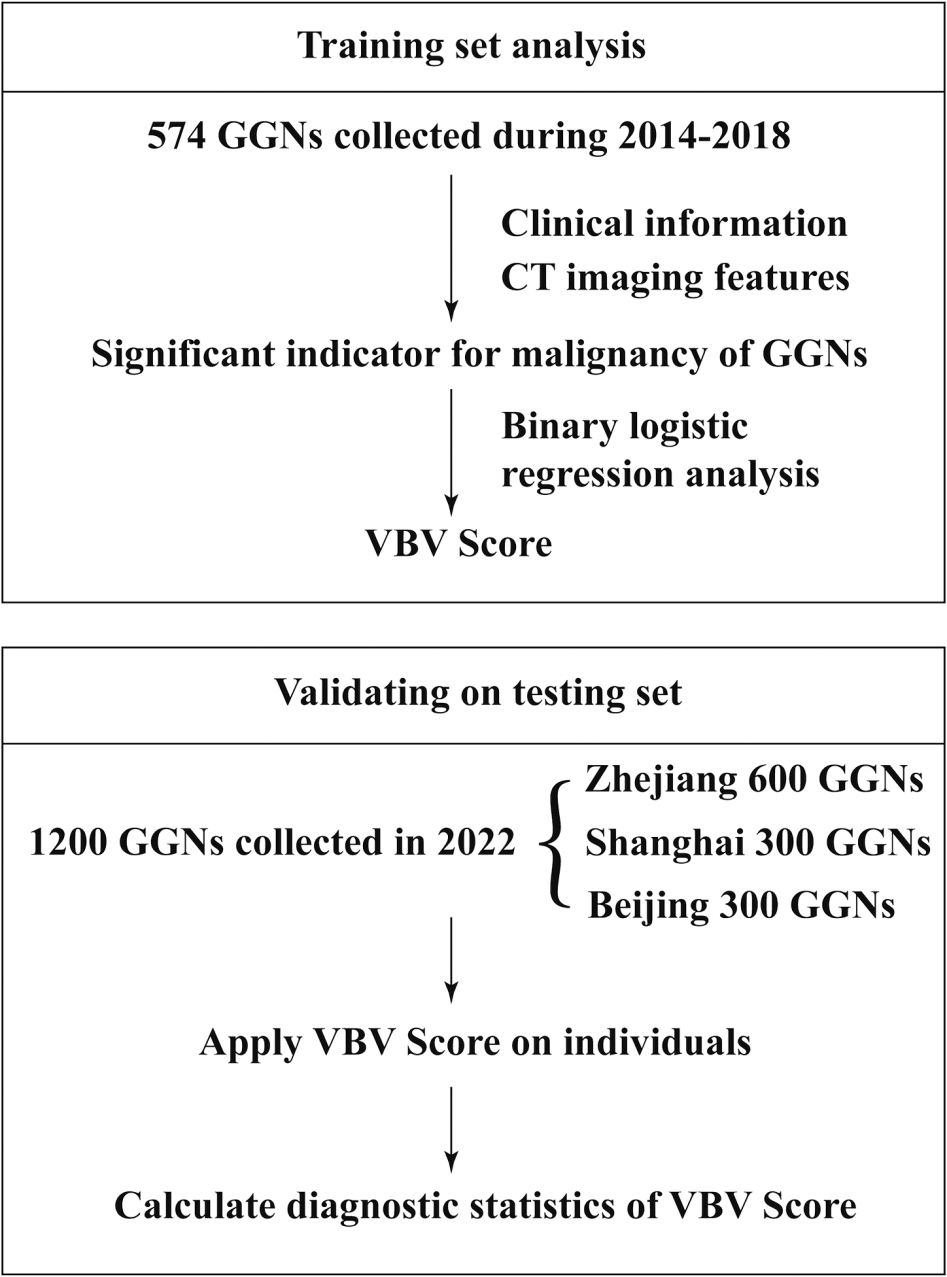
Establishment and validation of VBV Score for GGNs, GGNs: ground-glass nodules

**Fig. 2:**
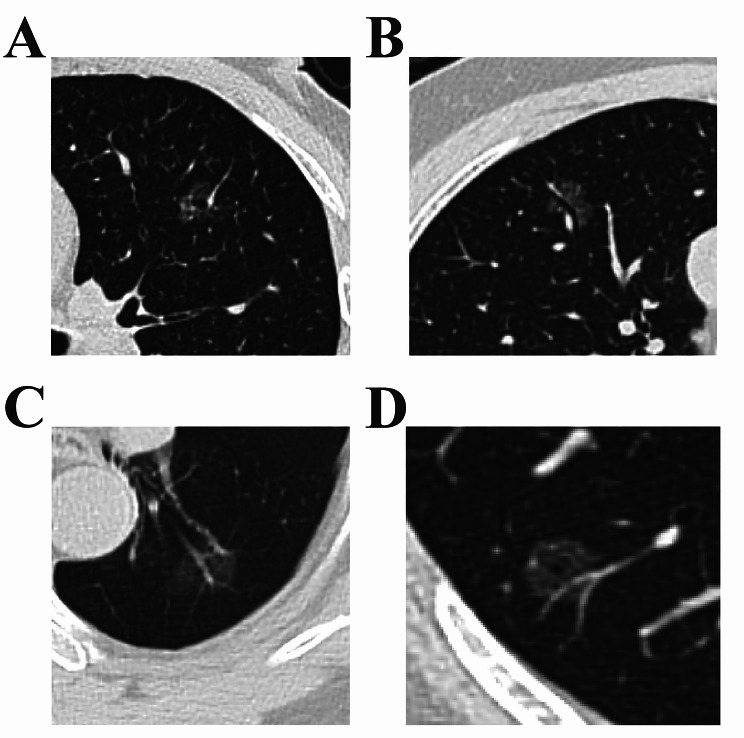
Image features of malignant GGNs in Axial HRCT. (**A**) Adenocarcinoma in a 55-year-old male. HRCT shows a 15 mm focal ground-glass nodule with vacuole sign sign and intra-nodular vessel sign in the left upper lobe. (**B**) Adenocarcinoma in a 57-year-old female. HRCT shows a 15 mm focal ground-glass nodule with air bronchogram sign and intra-nodular vessel sign in the right upper lobe. (**C**) Adenocarcinoma in a 67-year-old male. HRCT shows a 18 mm focal ground-glass nodule with intra-nodular vessel sign in the left lower lobe. (**D**) Adenocarcinoma in a 60-year-old male. HRCT shows a 18 mm focal ground-glass nodule with vacuole sign sign and intra-nodular vessel sign in the right lower lobe

According to the flow chart shown in Fig. 1 and 574 GGNs were included in the current study, which consist of 481(83.8%) malignant nodules and 93(16.2%) benign nodules (Supplementary Table 1). Table 1 showed clinical characteristics and CT features. Median patient age was 52 years old. The patients with malignant nodules were more likely to be women (*p* = 0.009, Table 1). There were no differences between malignant and benign GGNs with regard to age, follow-up time, smoking history, and histories of malignant tumors (*p* > 0.05, Table 1). The maximum diameter of malignant GGNs was larger than that of benign GGNs (0.865 ± 0.350 vs. 1.045 ± 0.360, *p* < 0.001, Table 1). Nearly 90% of the nodules showed maximum diameter of less than 1.5 cm. The frequencies of vacuole sign (12.9% vs. 67.6%, *p* < 0.001; Table 1; Fig. 2A), air bronchogram (1.1% vs. 21.6%, *p* < 0.001; Table 1; Fig. 2B), and intra-nodular vessel sign (11.8% vs. 78.6%, *p* < 0.001; Table 1; Fig. 2C、D) were higher in case of malignant nodules than in case of benign GGNs. Additionally, the malignant nodules were more likely to be located in an upper lobe than benign GGNs (*p* = 0.024, Table 1). There was no significant difference between benign and malignant GGNs with regard to other morphological characteristics, such as shape, lobulation, presence of spinous processes, pleural indentation, the occurrence of pleural effusion, pulmonary fibrosis, and emphysema (*p* > 0.05, Table 1).

**Table 1:** The clinical characteristics of 574 GGNs.

	Benign (*n* = 93)	Malignant (*n* = 481)	*p*-value
**Age(years)**	52.76 ± 9.754	52.59 ± 11.302	0.735
**Gender**			**0.009**
Male	40	141	
Female	53	340	
**Follow-up time**			0.098
Less than one month	27	102	
More than one month	66	379	
**Histories of malignant tumors**	6 (6.5%)	20 (4.2%)	0.330
**Smoking history**	20 (21.5%)	67 (13.9%)	0.062
**Maximum diameter of GGNs (mm)**	0.865 ± 0.350	1.045 ± 0.360	**< 0.001**
**Location**			**0.024**
**RUL**	28	176	
**RML**	9	33	
**RLL**	19	84	
**LUL**	17	134	
**LLL**	20	54	
**Shape**			0.370
**Round/oval**	61	338	
**Irregular**	32	143	
**Lobulation**	9 (9.7%)	62 (12.9%)	0.389
**Spinous process**	0	1 (0.2)	0.660
**Vacuole sign**	12 (12.9%)	325 (67.6%)	**< 0.001**
**Air bronchogram**	1 (1.1%)	104 (21.6%)	**< 0.001**
**Intra-nodular vessel sign**	11 (11.8%)	378 (78.6%)	**< 0.001**
**Calcification and fat**	0	0	/
**Necrosis**	0	0	/
**Pleural indentation**	6 (6.5%)	17 (3.5%)	0.189
**Pleural effusion**	0	0	/
**Emphysema**	2 (2.2%)	6 (1.2%)	0.496
**Pulmonary fibrosis**	0	0	/
**Other nodules**	28 (30.1%)	176 (36.6%)	0.232

### Binary logistic regression analysis and VBV score construction

In the binary logistic regression analysis, the gender (female), nodule location, maximum diameter, vacuole sign, air bronchogram, and intra-nodular vessel sign were regarded as independent variables. The results of the analysis showed that vacuole sign, air bronchogram, and intra-nodular vessel sign were important indicators of malignancy in GGNs (Table 2). The risk of malignancy for a GGN with vacuole sign, air bronchogram, and intra-nodular vessel sign was 16.651 times, 12.625 times, and 29.594 times higher, respectively, than that of a GGN without these characteristics. According to the obtained three significant variables and their coefficients, we derived a VBV Score as follows:

**Table 2:** Binary logistic regression analysis of GGNs

	Variables in the Equation							
							95 CI for OR	
	B	S.E.	Wald	df	Sig. (2-tailed)	Exp(B)	Lower	Upper
**Vacuole sign**	2.812	0.371	57.370	1	0.000	16.651	8.042	34.476
**Air bronchogram**	2.536	1.085	5.460	1	0.019	12.625	1.505	105.905
**Intra-nodular vessel sign**	3.388	0.375	81.813	1	0.000	29.594	14.204	61.659
Constant	-1.007	0.224	20.122	1	0.000	0.365		

S.E. = standard error

Wald = Wald chi-square value

df = degrees of freedom

Sig = significance

VBV Score = vacuole sign + air bronchogram + intra-nodular vessel sign (it added one point if the nodule had one of these characteristics; otherwise, none).

Then we established the Receiver Operating Characteristic Curve (ROC) According to the VBV score and calculated the Area Under the Curve (AUC) and Jorden index (Figure S1). If a nodule gets one point or more, it is considered a malignant nodule, otherwise it is a benign nodule. The sensitivity, specificity, and accuracy of this prediction model were 95.6%, 80.6%, and 93.2%, respectively (Table 3). This clinical model also showed great diagnostic concordance with a Kappa Coefficient of 0.753.

**Table 3:** Expected diagnosis of the malignancy of GGNs based on the VBV Score

Expected	Observed	
	Benign (*n* = 93)	Malignant (*n* = 481)
Benign	75 (80.6%)	21 (4.4%)
Malignant	18 (19.4%)	460 (95.6%)

Kappa = 0.753

### Validation of the VBV score using three independent cohorts of patients

Among the 1200 GGNs from three clinical centers, 1114 (92.8%) were diagnosed with malignancies, and detailed pathological Composition of GGNs was described in Supplementary Table 2. The three characteristics of VBV Score in each clinical center were listed in Supplementary Table 3, which showed that vacuole sign, air bronchogram, and intra-nodular vessel sign were still important indicators of malignancy in GGNs. We applied the VBV Score on these patients in same way. The sensitivity, specificity, and accuracy of this model for three validation set were 96.6%, 84.1%, 95.7%; 96.1%, 89.5%, 95.7%; and 94.6%, 82.6%, 93.7% respectively (Table 4). For all the 1200 GGNs, the sensitivity, specificity, and accuracy of this model were 96.0%, 84.9%, 95.2%. The Kappa Coefficient was 0.690.

**Table 4:** Diagnostic accuracy of the VBV Score in three independent cohorts of GGNs

	Observed							
	**Zhejiang**		**Shanghai**		**Beijing**		**Total**	
Expected	Benign (*n* = 44)	Malignant (*n* = 556)	Benign (*n* = 19)	Malignant (*n* = 281)	Benign (*n* = 23)	Malignant (*n* = 277)	Benign (*n* = 86)	Malignant (*n* = 1114)
Benign	37 (84.1%)	19 (3.4%)	17 (89.5%)	11 (3.9%)	19 (82.6%)	15 (5.4%)	73 (84.9%)	45 (4.0%)
Malignant	7 (15.9%)	537 (96.6%)	2 (10.5%)	270 (96.1%)	4 (17.4%)	262 (94.6%)	13 (15.1%)	1069 (96.0%)

Kappa (Zhejiang)=0.717

Kappa(Shanghai)=0.701

Kappa (Beijing)=0.633

Kappa (total)=0.69

## Discussion

In recent years, the widespread utilization of chest CT has prompted detection of pulmonary GGNs in otherwise asymptomatic individuals. For GGNs, we need to further judge whether they are benign or malignant. However, there is no uniform standard for judging GGNs, which is full of controversy. Clinicians often make judgments based on experience, which often leads to misdiagnosis of GGNs. Therefore, there is a growing need for a benign and malignant prediction model of pulmonary nodules.

There have been several studies that proposed pulmonary nodules predicting models by identifying some relative risk factors based on clinical and imaging features. Swensen et al. established Mayo Clinic model in 1997, which found that 3 clinical features (age, smoking history, history of previous malignancy) and 3 imaging features (nodule size, speculation, located in the upper lobe) were independent risk factors for malignant pulmonary nodules [16]. Gould et al. established a Veterans Affairs (VA) model using part of data from a multicenter VA study, including risk factors such as smoking history, older age, large nodule diameter, and time since quitting smoking [17]. Although these prediction models have a good prediction effect, the calculation process required is relatively complex, and a simple and easy to implement risk model is urgently needed in clinical practice for the differentiation of benign and malignant GGNs.

In the present study, our institution retrospectively collected and analyzed 574 GGNs from patients who were pathologically diagnosed. To ensure that the included pulmonary GGNs in the analysis have corresponding pathological diagnoses, for our analysis, we examined patients who underwent preoperative CT-assisted HOOK-WIRE puncture to locate nodules over the years. Our study demonstrated that vacuole sign, air bronchogram, and intra-nodular vessel sign in HRCT were associated with malignancy. According to the obtained three significant variables and their coefficients, we derived a simple scoring model (the VBV Score) for assessing pulmonary GGNs as follows:

VBV Score = vacuole sign + air bronchogram + intra-nodular vessel sign (it added one point if the nodule had one of these characteristics; otherwise, none).

If a nodule got at least one point, it was considered a malignant nodule, otherwise it was a benign nodule. This predictive model showed good sensitivity (95.6%), specificity (80.6%), accuracy (93.2%), and diagnostic concordance (the Kappa Coefficient = 0.753) in 574 GGNs and was validated via three independent cohorts of patients.

In our study, vacuole sign was more frequent in malignant GGNs than in benign GGNs (67.6% vs. 12.9%), which was probably caused by the lepidic tumor growth along the alveolar structure in adenocarcinomas. Several other reports have also revealed that the presence of vacuole sign is suggestive of adenocarcinoma [18, 19]. Besides, the frequency of air bronchogram was significantly higher in malignant GGNs than in benign GGNs (21.6% vs. 1.1%). F Wu. found air bronchogram could be helpful for distinguishing invasive pulmonary adenocarcinomas from pre-invasive lesions [20]. Intra-nodular vessel sign was essential for the malignancy of GGNs. As we all know, vascular invasion may promote tumor growth and metastasis, leading to recurrence or shortened survival in lung cancer patients [21]. The rate of malignant GGNs showing intra-nodular vessel sign was high (78.6%, 378/481); however, this characteristic was also observed in 11.8% (11/93) of benign nodules. Our findings are consistent with C Lin’s study that the infiltration of pulmonary arteries into lung lesions could indicate a higher blood supply, which may be conducive to the growth of malignant tumors [22].

**Fig. 3:**
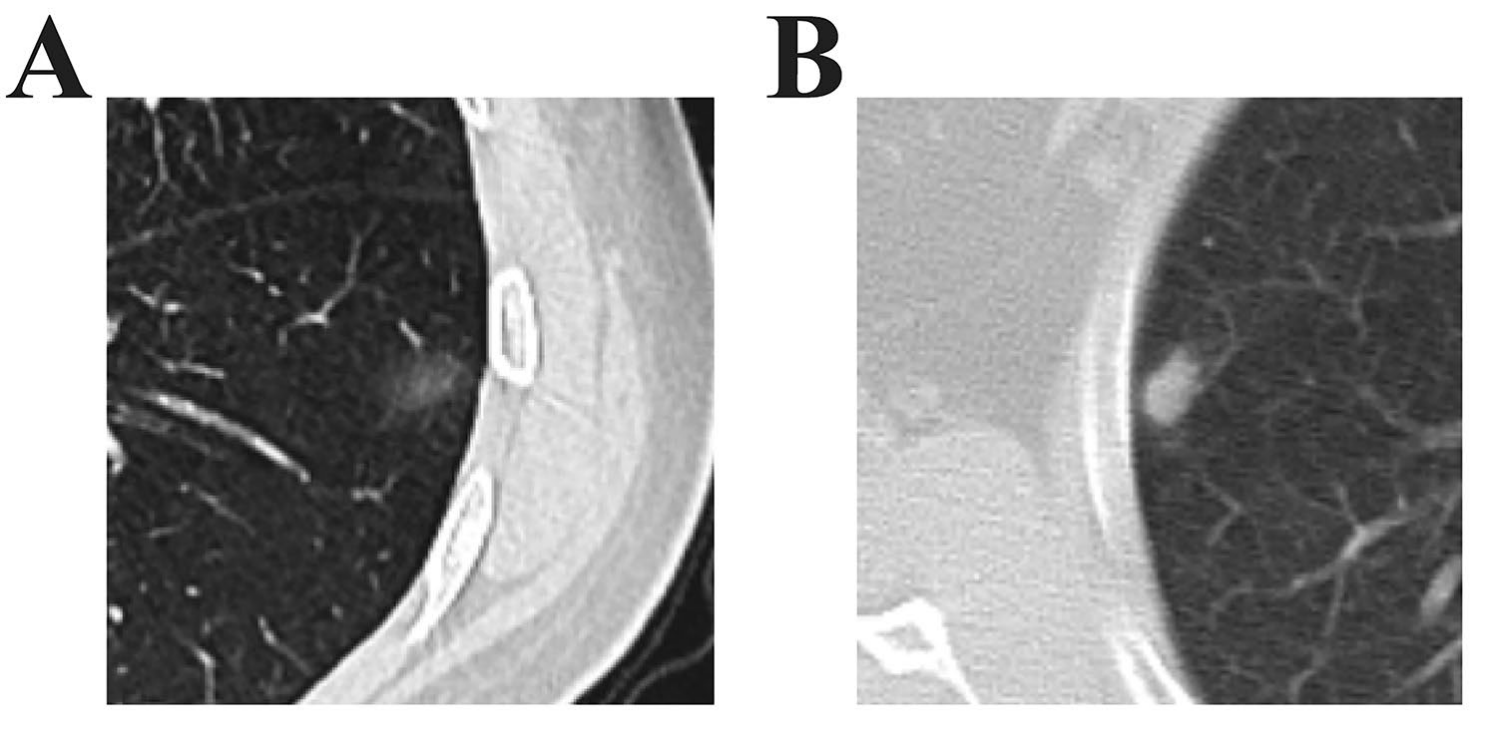
Image features of benign GGNs in Axial HRCT. (**A**) Chronic pneumonia in a 47-year-old female. HRCT shows a 14 mm focal ground-glass nodule with cloudy sign in the left lower lobe. (**B**) Granulomatitis with necrosis in a 63-year-old female. HRCT shows a 12.3 mm focal ground-glass nodule with homogenized patch sign in the right upper lobe, the mean CT attenuation value of nodule was − 221.90HU.

However, lobulation, spiculated margin, and pleural indentation were not significantly associated with malignant GGNs in this study; which was inconsistent with previous studies [23]. Most of the GGNs we collected were 1–2 cm in size, which mean lobulation and spiculation were relatively inconspicuous in these small nodules. Y Silva reported that with the increase in nodule diameter, the probability of the nodules being malignant also increases [24]. Although the results of our study indicated that malignant GGNs showed a markedly larger diameter than benign nodules, the nodule diameter was not significant with regard to the malignancy status of the nodules, as revealed by the logical regression analysis. On the other hand, for the nodules with pathological diagnosis of inflammation, lymph nodes or benign tumor, they may present as homogeneous patches, cloudy sign rather than ground-glass appearance (Fig. 3A、B). Usually, the pathology of homogenized patch nodules is specific to benign tissues, such as lymph nodes or hamartomas [25]. Cloudiness is another indication that the nodule is benign and presents as a blurred boundary. Nodules presenting as homogenized patch shadows and cloudy sign lacked the significant features mentioned above and probably absorbed after several months.

The purpose of the present study is to establish a score model that allows inexperienced clinician to make a preliminary judgment regarding GGNs on the basis of CT imaging features. Based on the VBV Score, if a pulmonary GGN got at least one point, in other words, had at least one morphological characteristic we describe, we considered that the GGN be malignant. On the contrary, if the score is zero, the GGN is likely to be benign. In order to facilitate the popularization and application of this scoring model, we named it the VBV Score. Features included in this study such as vacuole sign, air bronchogram, and intra-nodular vessel sign are easier to observe than well-defined margin, lobulation and speculation sign for patients with early-stage lung cancer, which may reduce the error caused by subjective judgment. Besides, the specificity performs good (80.6%), while our model has high sensitivity (95.6%), which is better than previous GGN predictive models with a sensitivity of 93.4% and a specificity of 66.7% [26]. For GGNs, improving specificity and reducing false-positive patients is particularly important, which can avoid overtreatment. For patients who have not been diagnosed with malignant nodules, as long as they are regularly followed up, treatment will not be delayed.

However, there are still several limitations. First, this is a multi-center retrospective study. All the cases we collected were considered to be malignant nodules and required pulmonary surgery after a period of follow-up, making the majority of benign cases already screened out. Moreover, it may increase the proportion of benign nodules that all the 574 patients we analyzed underwent preoperative CT-assisted HOOK-WIRE puncture to locate nodules. Both of these can lead to selection bias. Another limitation is that while the scoring model established in this study is accurate, we should recognize that, for now, the model can only be used as an auxiliary tool to help assessing GGNs. In the future, we hope to conduct a prospective multicenter study to further verify the value of VBV Score in the differential diagnosis of benign and malignant GGNs.

## Conclusions

The vacuole sign, air bronchogram, and intra-nodular vessel sign were important indicators of malignancy in GGNs. Then we constructed and validated the VBV Score model for assessing pulmonary GGNs as follows: VBV Score = vacuole sign + air bronchogram + intra-nodular vessel sign. This predictive model showed good sensitivity (95.6%), specificity (80.6%), accuracy (93.2%), and diagnostic concordance (the Kappa Coefficient = 0.753), which can help clinicians assess the risk of GGNs and make clinical decisions.

### Electronic supplementary material

Below is the link to the electronic supplementary material.

Supplementary Material 1

## Data Availability

The datasets used during the current study are available from the corresponding author on reasonable request.

## Abbreviations

HRCT: High-resolution computed tomography
GGNs: Ground-glass nodules
DICOM: Digital Imaging and Communications in Medicine
RUL: Right upper lobe
VATS: Video-assisted thoracic surgery
LR: Likelihood ratio
VA: Veterans Affairs
