# Supplementary material for: Establishing and validation of the VBV score for assessing Lung ground-glass nodules based on high-resolution computed tomography
Source: J Cardiothorac Surg. 2024 Jan 23;19:17. doi: 10.1186/s13019-024-02487-3 (PMC10804577; doi:10.1186/s13019-024-02487-3)
Supplement: Supplementary file 1 — Supplementary Material 1 [file 13019_2024_2487_MOESM1_ESM.docx]

According to the VBV score established in this study, 574 cases of ground glass nodules(GGO) with pathological diagnosis were scored. We established the Receiver Operating Characteristic Curve (ROC) based on the scores obtained (Figure S1) and calculated the Area Under the Curve (AUC) and Jorden index in order to select the optimal threshold point. The results showed that AUC=0.912, Jorden index =0.762, and the optimal threshold point T=0.5, which means the pulmonary glass nodule (GGO) was considered to be malignant while the VBV score was greater than or equal to 1; the pulmonary glass nodule (GGO) was considered to be benign while the VBV score was 0.


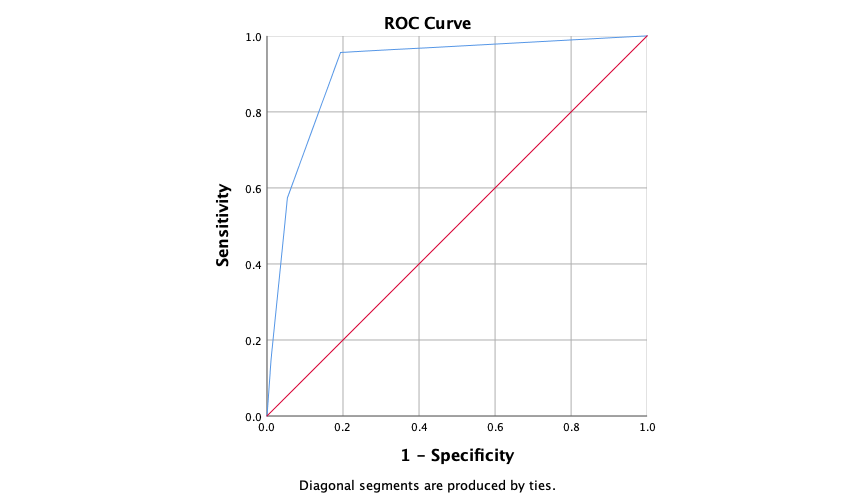


**Figure S1**

**Supplement Table 1 Pathological Composition of 574 GGNs during 2014-2018.**

| **Histologic Type and Subtypes** | **No. Patients** |
| --- | --- |
| **Preinvasive lesions** |  |
| atypical adenomatous hyperplasia | 32 |
| adenocarcinoma in situ | 27 |
| **Minimally invasive adenocarcinoma** | 131 |
| **Invasive non-mucinous adenocarcinoma** | 288 |
| **Invasive mucinous adenocarcinoma** | 2 |
| **Entric-type adenocarcinoma** | 1 |
| **Hamartoma** | 4 |
| **Lymph nodes** | 20 |
| **Lung tissue hyperplasia** | 15 |
| **Angiomatous hyperplasia** | 7 |
| **Fibrotic nodules** | 5 |
| **Inflammation** | 32 |
| **Granulomatitis** | 16 |
| **Cryptococcosis** | 1 |

Note: Tumor types and subtypes according to the 2011 IASLC/ATS/ERS and 2015 WHO classifications.

**Supplement Table 2 Pathological Composition of GGNs from three clinical centers.**

| **Histologic Type and Subtypes** | **Zhejiang** | **Shanghai** | **Beijing** |
| --- | --- | --- | --- |
|  | GGNs  No. Patients | GGNs  No. Patients | GGNs  No. Patients |
| **Precursor glandular lesions** |  |  | 5 |
| atypical adenomatous hyperplasia | 18 | 7 |  |
| adenocarcinoma in situ | 50 | 32 | 18 |
| **Adenocarcinomas** |  |  |  |
| microinvasive adenocarcinoma | 366 | 116 | 102 |
| invasive non-mucinous adenocarcinoma | 269 | 127 | 149 |
| invasive mucinous adenocarcinoma | 8 | 3 | 3 |
| **Squamous cell carcinomas** |  | 0 | 2 |
| **Hamartoma** | 7 | 3 | 0 |
| **Lymph nodes** | 11 | 3 | 1 |
| **Lung tissue hyperplasia** | 13 | 5 | 4 |
| **Fibrotic nodules** | 4 | 1 | 2 |
| **Inflammation** | 22 | 3 | 17 |
| **Granulomatitis** | 9 | 3 | 1 |

Note: Tumor types and subtypes according to the 2021 World Health Organization classification scheme.

**Supplement Table 3 Three characteristics of Hangzhou Score in three independent clinical centers.**

|  | **Zhejiang** | |  | **Shanghai** | |  | **Beijing** | |  |
| --- | --- | --- | --- | --- | --- | --- | --- | --- | --- |
|  | Benign  (n=44) | Malignant  (n=556) | p-value | Benign  (n=19) | Malignant  (n=281) | p-value | Benign  (n=23) | Malignant  (n=277) | p-value |
| **Air cavitation** | 5 (12.2%) | 334 (62.9%) | **<0.001** | 0 | 122 (43.4%) | **<0.001** | 3 (13.0%) | 204 (73.6%) | **<0.001** |
| **Air bronchograms** | 1 (2.5%) | 55 (11.0%) | 0.089 | 0 | 14 (5.0%) | 0.319 | 2 (8.7%) | 105 (37.9%) | **0.005** |
| **Intra-nodular vessel sign** | 6 (15.0%) | 444 (83.5%) | **<0.001** | 2 (10.5%) | 243 (86.5%) | **<0.001** | 1 (4.3%) | 224 (80.9%) | **<0.001** |
